# Multiple erythroid isoforms of human long-chain acyl-CoA synthetases are produced by switch of the fatty acid gate domains

**DOI:** 10.1186/1471-2199-7-21

**Published:** 2006-07-11

**Authors:** Eric Soupene, Frans A Kuypers

**Affiliations:** 1 Children's Hospital Oakland Research Institute, Oakland, California 94609, USA

## Abstract

**Background:**

The formation of acyl-CoA by the action of acyl-CoA synthetases plays a crucial role in membrane lipid turnover, including the plasma membrane of erythrocytes. In human, five Acyl-CoA Synthetase Long-chain (*ACSL*) genes have been identified with as many as 3 different transcript variants for each.

**Results:**

Acyl-CoA Synthetase Long-chain member 6 (ACSL6) is responsible for activation of long-chain fatty acids in erythrocytes. Two additional transcript variants were also isolated from brain and testis. We report the expression in reticulocytes of two new variants and of the one isolated from brain. All three represented different spliced variants of a mutually exclusive exon pair. They encode a slightly different short motif which contains a conserved structural domain, the fatty acid Gate domain. The motifs differ in the presence of either the aromatic residue phenylalanine (Phe) or tyrosine (Tyr). Based on homology, two new isoforms for the closely related ACSL1 were predicted and characterized. One represented a switch of the Phe- to the Tyr-Gate domain motif, the other resulted from the exclusion of both. Swapping of this motif also appears to be common in all mammalian ACSL member 1 and 6 homologs.

**Conclusion:**

We propose that a Phe to Tyr substitution or deletion of the Gate domain, is the structural reason for the conserved alternative splicing that affects these motifs. Our findings support our hypothesis that this region is structurally important to define the activity of these enzymes.

## Background

In mammals, long-chain acyl-CoA synthetases (ACSL) are necessary for fatty acid degradation, phospholipid remodeling, and production of long acyl-CoA esters that regulate various physiological processes. These enzymes play a crucial role in plasma membrane phospholipid turnover in erythrocytes, via the Lands pathway [1], as these cells lack the capacity for *de novo *lipid synthesis.

In human, five *ACSL *genes have been identified with as many as 3 different transcript variants for each. The two recognized transcript variants for *ACSL6 *(formerly *LACS5 *[2]) were the only ones reported that represented spliced forms of a mutually exclusive pair of exons encoding a short highly conserved motif in the middle of the protein. All other variants differed in the amino terminus and/or the 5' UTR region. Whereas the detailed structure of mammalian ACSL has not been reported, the crystal structure of a bacterial homologue of ACSL has established this motif at the proximity of the catalytic site of the enzyme, defining the entry gate for the fatty acid substrate [3]. This region, referred to as the Gate domain, is also involved in the formation of a pocket, the "dead-end branch", in which the fatty acid is locked during the formation of the CoA ester bond [3]. The depth and width of the pocket likely defines the substrate specificity of each of the isoforms. In the bacterial homologue, a tryptophan residue of the Gate domain blocks the fatty acid channel and prevents entry of the acyl to the catalytic site. Binding of ATP to the nearby P-loop results in rotation of this residue and opening of the channel [3]. Interestingly, mammalian ACSLs do not have this tryptophan residue and another aromatic residue, tyrosine or phenylalanine, is conserved at a different position.

For human ACSL6, although the region containing the Gate domain is alternatively spliced, the Gate domain itself is conserved. In both variants a consensus sequence can be defined as D-x_4_-(Y, F)-LPLAH-x_2_-E, and we postulated that the substitution of a Y (variant 1) to F (variant 2) residue underlies the reason for the alternative splicing of the Gate-domain region. In addition to the variant originally found in erythroid cells, ACSL6_v1, we now have isolated 3 additional variants from cDNA of reticulocytes: the spliced variant originally found in brain, ACSL6_v2, and two new isoforms, ACSL6_v4 and v5. We also identified a fifth variant, ACSL6_v3, present in the GenBank database, which had not previously been recognized as a different spliced transcript. Based on protein similarity between different members of the ACSL family we hypothesized that the only known isoform of ACSL1 was in fact one of the two possible versions of the Gate domain. Indeed we were able to identify and isolate from different tissues two new transcripts representing spliced variants of this domain, ACSL1_v1 and v3. We provide evidence that the two versions of the Gate domain, which we define as the Y- or F-Gate depending on the Y to F residue substitution, are evolutionary conserved. These different Gate versions are present in amphibian, fish, fly, bird and plant. They can be the product of separate genes in some organisms, e.g. plants, or be obtained by an alternative switching affecting ACSL1 and 6 in others, e.g. mammals.

## Results

### Five isoforms of ACSL6

**Figure 1:**
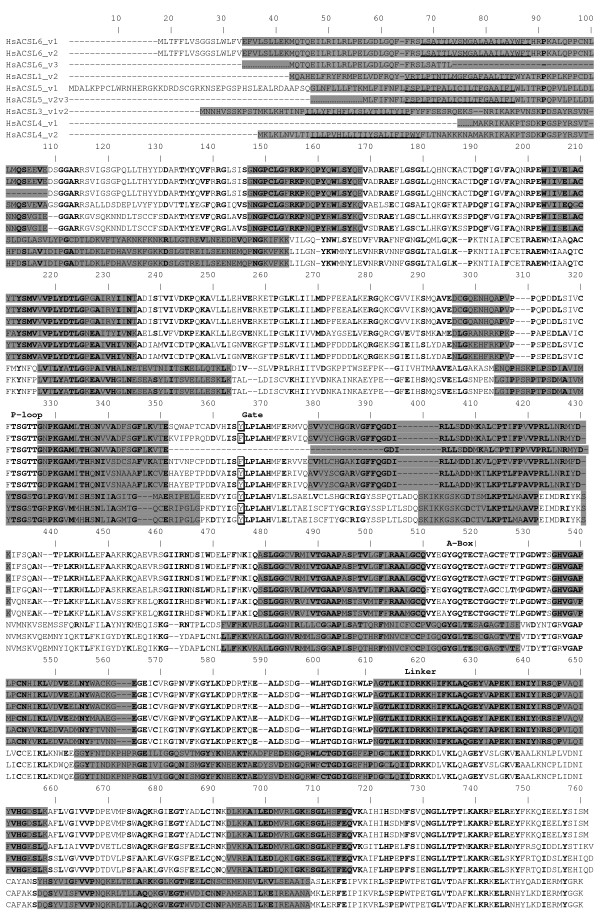
**Sequence alignment of the human acyl-CoA synthetases long-chain family members**. The succession of clear-shaded blocks overlaid on the alignment represents the exon organization of the respective mRNA. Conserved residues are in bold. Position of the P-loop, Gate, A-box and Linker motifs is indicated. Y and F residues of the two alternative Gate domains are boxed and indicated with a star. Transmembrane spanning segments predicted with the highest score by TopoPrediction, SOSUI and TMHMM are underlined. For clarity, ACSL3 variant 1 and 2, and ACSL5 variant 2 and 3, which encode the same isoform, are annotated as ACSL3_v1v2 and ACSL5_v2v3, respectively. Alignment was performed with MUTALIN [14]. Protein length (residues): ACSL1_v1, 698; ACSL1_v2, 698; ACS3_v1v2, 720; ACSL4_v1, 670; ACSL4_v2, 711; ACSL5_v1, 739; ACSL5_v2v3, 683; ACSL6_v1, 722; ACSL6_v2, 722; ACSL6_v3, 622. GenBank accessions numbers are given in Table 2.

**Figure 2:**
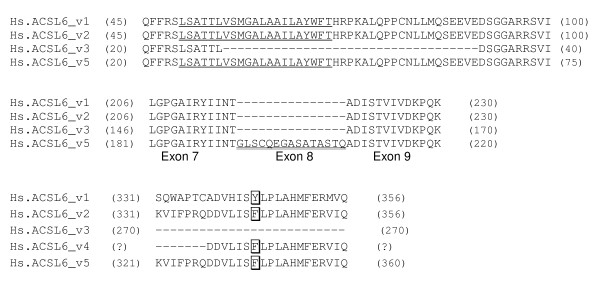
**Details of the sequence differences of the five human ACSL6 isoforms**. The Y and F residues of the Gate domains are boxed. Position of the first and last residues shown is indicated on the left and right, respectively. Note that only the Gate-domain region of ACSL6_v4 is known. A predicted transmembrane spanning segment at the amino-terminus is shown underlined. The inserted sequence in between Exon 7 and Exon 9 of variant 5, annotated as Exon 8, is shown double-underlined.

ACSL6 variant 1 and variant 2 (ACSL6_v1 and ACSL6_v2) represent spliced variants of two mutually exclusive exons (Figures 1, 2 and 5). Both exons code for a short motif of 26 residues which contain a highly conserved domain in the ACSL family; D-x_4_-(F, Y)-LPLAH-x_2_-E (Figures 1 and 5B). In the description of the structure of the bacterial homologue of ACSL, this region was shown to contain an entry gate for the fatty acid substrate [3]. Therefore, we will refer to this region as the F- or Y-Gate domain which contains either a F or Y and the two exons as Ex(F) and Ex(Y), respectively. ACSL6_v1 was originally isolated from K562 cells [2] and ACSL6_v2 from brain [GenBank:AB020644.1]. We isolated both isoforms from several other tissues (Table 1).

We identified an additional variant form of *ACSL6*, as ACSL6_v3 [GenBank:BC047453.1]. ACSL6_v3 lacks the first coding exon and represents a truncated amino terminus isoform. An alternative 3'-splice site appears to be present in exon 3 as it also lacks the second half of exon 3 (Figures 1 and 2). This alternative splicing event results in removal of a predicted membrane spanning segment. Moreover, both Ex(F) and Ex(Y) are skipped in this transcript variant which results in a product without the Gate domain.

Two new variants of *ACSL6 *were identified that we propose to name ACSL6_v4 and ACSL6_v5 (Table 1). Both were isolated from reticulocyte cDNA and they represent spliced variants of ACSL6_v2 (Figures 2 and 5). ACSL6_v4 lacks the first half of Ex(F) which is the least conserved region among all variants (Figures 2). The actual Gate domain is encoded by the second half of either Ex(F) or Ex(Y). Whereas a similar variation may be expected in Ex(Y) of ACSL6_v1, we were not able to detect a truncated version of Ex(Y) in a survey of 98 clones (see Methods). Ex(F) appears to contain an alternative 3' acceptor splicing site.

ACSL6_v5 contains an additional sequence of 45 bases between exon 7 and 8 as compared to ACSL6_v2 (Figures 2 and 5). This element perfectly matched a portion of intron 7 in the genomic sequence (NT_034772.5, position 33739262–33739306). The insertion occurs at the 5' donor splicing site of exon 7, position +1 of the last codon, (E7)-G/CG-(E9). This event results in the in-frame translation of an additional 15 residues at position T^192 ^(Figure 2) and restoration of the GCG codon, (E7)-**G**/GG-new exon-G\**CG**-(E9). We propose that this element constitutes a new exon, exon 8.

Altogether, we have newly identified or confirmed five different forms of ACSL6 (Table 2) expressed by alternative splicing as summarized in Figure 5.

### ACSL6 forms dimers in the plasma membrane

As already mentioned, ACSL6_v3 lacks the putative amino-terminal transmembrane segment that was predicted by several different analysis tools. Various topology tools predict up to three transmembrane segments of ACSL6, but most of these models are not consistent with predictions for the other ACSL isoforms and are probably not compatible with the domain organization of the protein. Based on the activity of the enzyme, both the ATP-binding site and the C-terminus domain need to face the cytosol. However, in several predictions, these regions would be exposed to the wrong and/or opposite sides of the membrane.

**Figure 3:**
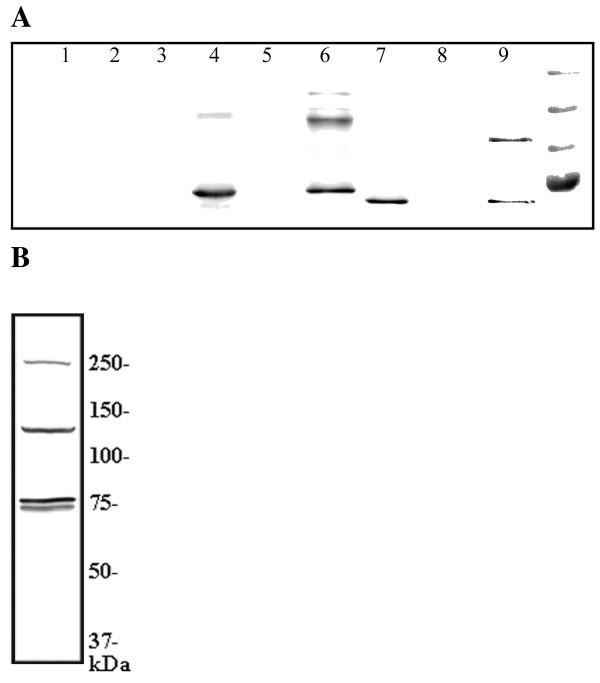
**Detection of ACSL6 isoforms in *E. coli *(A) and in human K562 cells (B)**. **A: **vector (1, 2, 3); ACSL6_v2 (4, 5, 6); ACSL6_v3 (7, 8, 9). Crude extract (1, 4, 7); Soluble fraction (2, 5, 8); Membrane fraction (3, 6, 9). Detection performed with Pierce INDIA HIS-antibody. Protein ladder standard is shown on the right (top to bottom: 250-150-100-75 kDa). Proteins carried a hexahistidine-tag at their amino-terminus (see Methods). **B: **Total protein extract of K562 cells. Detection performed with a peptide raised antibody against ACSL6.

ACSL6_v1, v2 and v3 were cloned and expressed as hexahistidine fusion proteins in *E. coli *(see Methods). Despite that the predicted structure precludes a proper insertion of variant 3 in a membrane lipid bilayer, product of variant 3 was found in a membrane fraction, as was v1 and v2 product (Figure 3A and data not shown). It can be argued that ACSL6_v3 was not inserted but associated with the membrane as was suggested for the *E. coli *FadD homolog [4] and rat ACSL4 [5]. Alternatively, another region of the protein may have assured anchoring to the membrane.

When analyzed on denaturing SDS polyacrylamide gel (see Methods), each of the three isoforms appeared as two species of apparent molecular mass of 75–80 kDa and 130 kDa (Figure 3A and data not shown). A slightly lower apparent molecular mass was observed for the shorter variant ACSL6_v3. The predicted molecular mass was 80 kDa for variants 1 and 2 and 72 kDa for v3. Thus, as it has been established for the bacterial homologue [3], human ACSL6 appeared to be a dimer. With a protein extract of the erythroleukaemic cell line K562, an ACSL6 antibody recognized three major species at 75 kDa and at 130 kDa (Figure 3B). The two bands migrating at the expected position for a monomer probably represent isoforms of slightly different molecular mass (Table 1).

### Identification of new *ACSL1 *transcript variants

By sequence inspection of the Gate-domain motifs, we noted that only two isoforms of ACSL, ACSL1 and ACSL6_v2, of the 8 known isoforms (ACSL1, ACSL3, ACSL4_v1 and _v2, ACSL5_v1 and v2/v3; ACSL6_v1 and _v2) had an aromatic residue F instead of a Y (Figure 1). We also noted that alternative splicing of ACSL6 resulted in a F to Y substitution (Figures 2 and 5). Given that the other conserved residues were not affected by this event, we predicted the presence of spliced variants of ACSL1.

**Figure 4:**
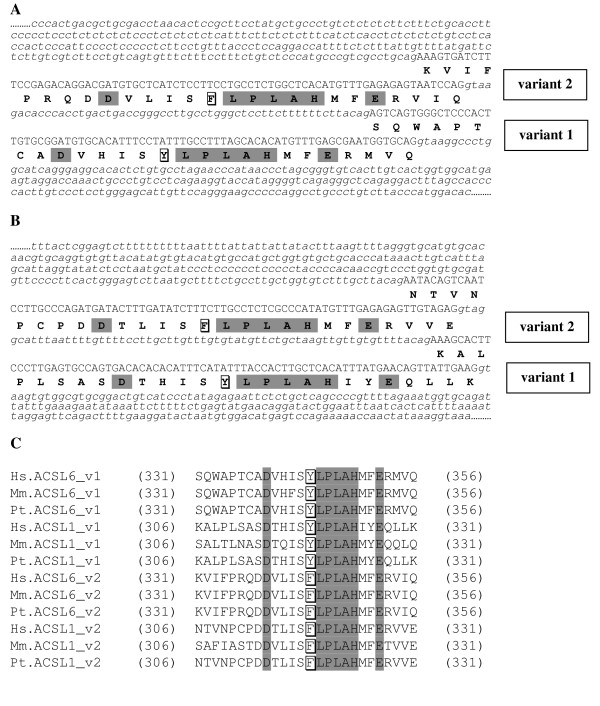
**Genomic and amino acid sequences of the gate-domain region of human *ACSL6 *and *ACSL1***. **A and B: **Sequence of exons and introns of human *ACSL6 *(A) and *ACSL1 *(B) are represented by capitalized and small italics letters, respectively. The F and Y residues of the Gate domain of variant 2 and 1, respectively, are boxed. **C: **Amino acids alignment of the Gate-domain motifs for known and predicted spliced variants. Highly conserved residues are shaded. Position of the first and last residues shown is indicated on the left and right, respectively. Abbreviations are: Hs, Homo sapiens; Ms, Mus musculus; Pt, Pan troglodytes.

**Figure 5:**
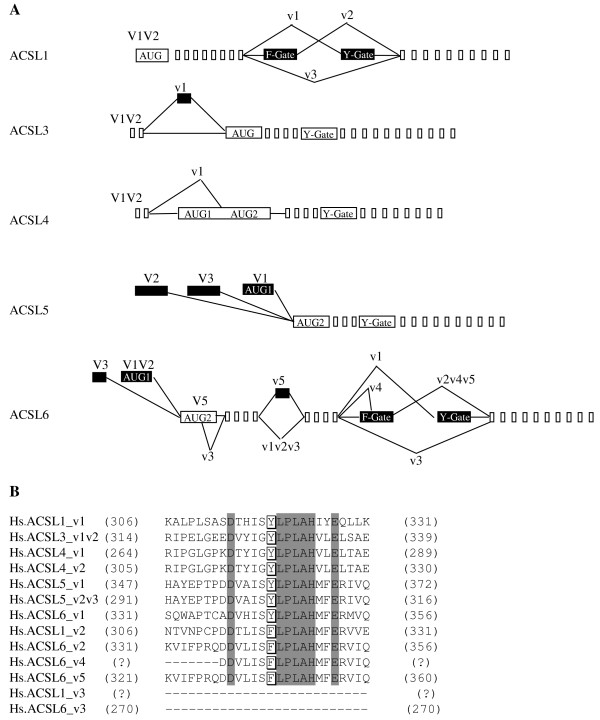
**Human *ACSL *transcript variants (A) and gate domains (B)**. **A: **Schematic representation of the different known transcript variants. Exons are represented by empty boxes and alternative exons by filled boxes. Translation initiator AUG and Y/F-Gate domain encoding exons are indicated. If known, a capitalized V indicates the first exon of each variant. Note that only the sequence of the Gate-domain region of ACSL1_v3 and ACSL6_v4 is known. See Results for details. **B: **Alignment of the Gate-domain motifs. The F and Y residues of the two Gate domains are boxed. Highly conserved residues are shaded. Note that more residues are conserved among human ACSL isoforms. Position of the first and last residues shown is indicated on the left and right, respectively. Note that only the Gate-domain region of ACSL1_v3 and ACSL6_v4 is known. For clarity, ACSL3 variant 1 and 2, and ACSL5 variant 2 and 3, which encode the same isoform, are annotated as ACSL3_v1v2 and ACSL5_v2v3, respectively.

In human, mouse, and chimpanzee the exon encoding the Y-Gate domain of ACSL6_v1 is downstream of the exon encoding the F-Gate domain of ACSL6_v2 (Figure 4 and not shown). The only reported ACSL1 isoform contains the F-Gate domain, coded by exon 10. We identified a potential new exon encoding a Y-Gate domain in the intronic sequence downstream of *ACSL1 *exon 10 of human, and exon 11 of mouse and chimpanzee (Figure 4 and not shown). As described in Methods, we surveyed the Gate-domain motifs of human *ACSL1 *by PCR cDNAs from various tissues. In addition to the known ACSL1 variant containing the F-Gate domain, re-annotated ACSL1_v2, we identified a variant with a Y-Gate domain in placenta and K562 cells, ACSL1_v1, and a variant without a Gate domain in CD34 positive cells and K562 cells, ACSL1_v3 (Table 1 and Figure 5). From sequence inspection, it appeared that the other three ACSL members, ACSL3, 4 and 5 can only encode a Y-Gate domain (see Discussion).

### The fatty acid gate domains of human ACS

**Figure 6:**
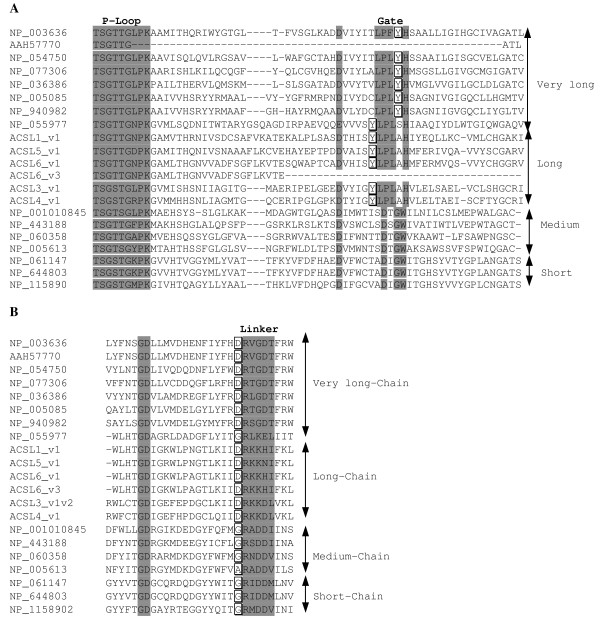
**Gate (A) and linker (B) domains of human acyl-CoA synthetase family members**. The Y residue of the Gate domain for long and very-long-chain ACS is boxed. The D and G residues of the Linker domains are boxed. GenBank accession numbers for short, medium, long and very-long chain are indicated on the left. The Gate domain of BG1 is of the long-chain type but its Linker is of the short-medium type. Lipidosin is a soluble ACS very-long-chain isoform found in brain tissue and is known to have a broader substrate range than other very-long ACS [17, 18].

**Figure 7:**
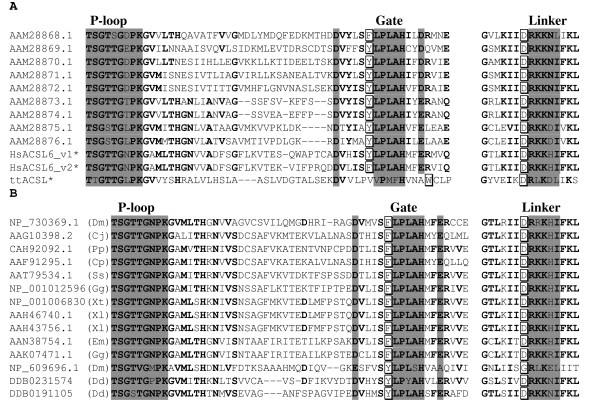
**Conservation of the gate and linker motifs among ACSL homologs**. **A: **Alignment of the nine Arabidopsis ACSL forms. Human ACSL6 spliced variant 1 and 2, and ACSL of *Thermus thermophilus *(ttACSL) indicated with an asterisk (*) are shown as references in the last three rows. **B: **Alignment of various forms of ACSL from different organisms. Abbreviations: Dm, Drosophila melanogaster (fly); Cj, Callithrix jacchus (marmoset); Pp, Pongo pygmaeus (orangutan); Cp, Cavia porcellus (guinea pig); Ss, Sus scrofa (pig); Gg, Gallus gallus (chicken); Xt, Xenopus tropicalis (frog); Xl, Xenopus laevis (frog); Em, Eleginops maclovinus (fish); Gg, Gobionotothen gibberifrons (fish); Dd, Dictyostelium discoideum (slime mould).

An amino acid sequence comparison of the four different families of human acyl-CoA synthetase proteins (Short, Medium, Long and Very-long chain) revealed major differences in the Gate-domain motifs. A consensus motif for Short and Medium chain appears to be D-x6-D-x-G-W, in which W might represent the gating residue (Figure 6). Long and Very-long chain ACS shared the Gate-domain motif D-x_5_-LPL-x-H in common but the conserved Y residue (F for some variants) found at the -1 position of the LPL-x-H motif for Long chain, **Y**-LPL-x-H, is shifted to another position for the Very-long chain ACS, x-LPL-**Y**-H. The conserved Linker motif [6] also supports the division between Short-Medium and Long-Very-long. Long and Very-long ACS, with the exception of human lipidosin or bubblegum (BG1; see footnote Figure 6), contain an Aspartate residue while the Short and Medium ACS have a Glycine. As previously proposed [3], substitution of an Asp to a Gly might introduce enough flexibility in this region to allow two different conformations of the C-terminus domain. Of the eleven putative ACS long-chain plant homologues, two had no detectable activity and were excluded from the ACS long-chain plant family [7]. Although all eleven showed a very high degree of similarity to each other, only the nine active isoforms have Gate and Linker consensus motifs for long-chain (Figure 7). The other two lack an aromatic residue at the -1 position of the LPL-x-H motif and had a Gly residue instead of an Asp in their Linker motif.

## Discussion

The mechanism by which Short, Medium, Long and Very-long chain acyl-CoA synthetases achieve substrate specificity is poorly understood. In spite of their high degree of similarity and highly conserved structural domains, length and degree of un-saturation of the fatty acid substrates are being discriminated. Of particular interest is a specific functional region in the bacterial protein that contains the so called Gate domain [3]. In absence of ATP, an aromatic residue, tryptophan, of the Gate domain blocks the fatty acid tunnel and as such, the access of the fatty acid to the active site. Upon binding of ATP to the nearby P-loop, a conformational change induces rotation of this residue and the opening of the channel. The hydrophilic head group of the fatty acid reaches the catalytic site while the hydrophobic tail is bent into the dead-end branch. The depth and width of the pocket formed by the Gate domain is a tight fit for the preferred substrates of the enzyme [3]. The tryptophan is not conserved in mammalian ACSL. As described in this study, expression of different Gates with substitutions of an aromatic residue (tyrosine to phenylalanine) might achieve different gating and selectivity properties toward different substrates. Measure of the substrate specificity of those membrane-bound enzymes is complicated by differences in substrate availability of the different fatty acids in the various pools in the lipid bilayer, and by the necessary utilization and/or removal of the acyl-CoA product (which is inhibitory and detergent in nature). Although enzyme activities have been assessed in *E. coli*, we found that both human and rat ACSL6 accumulated in inclusion bodies when over-expressed. The small fraction of proteins recovered in the lysate was purified to various degrees and assayed in Triton X-100 micelles [8]. While we can measure acyl-CoA formation of isoforms expressed in *E. coli *we refrain from defining enzymatic activity (Km and Vmax) for different fatty acids in this system, as these data would not necessarily reflect the specificity of these isoforms in the mammalian plasma membrane, and therefore not add to a better understanding of the actions of these proteins. The activated fatty acyl groups generated in the red cell membrane by ACSL6 are bound or processed by the other proteins in the Lands pathway affecting the activity of ACSL6. In addition, lipid-protein interactions will affect the activity of these isoforms precluding the determination of proper enzyme kinetics in an artificial detergent rich lipid environment as reported [8]. Therefore, we argue that enzymatic characterization of the different isoforms, homo- and hetero-dimers, in the plasma membrane with proper lipid/protein and, lipid/lipid interactions affecting enzyme activity will ultimately be essential and necessary to understand the selectivity of this process in lipid and protein acylation. The characterization of the different isoforms present in a plasma membrane defines the first step in this process.

The validity to compare the bacterial homologue with the human forms is supported by the evolutionary conservation of the Gate-domain motif in invertebrates and vertebrates (Figure 7). In mammals, two members, ACSL member 1 and 6, can produce the two different Gate domains by alternative splicing. Sequence inspection of the other three *ACSL *genes, *ACSL3*, *4 *and *5*, strongly supports the prediction that they can only encode one type of Gate domain, Y-Gate. Thus, the F-Gate is specific for *ACSL1 *and *6*. The conservation of the aromatic residue, Phe or Tyr, in all forms of ACSL supports our hypothesis that this region is structurally important to define the activity of these enzymes [9].

ACSL1 mRNA was highly expressed in erythroid progenitors, CD34 positives cells, but it could not be detected by Northern-blot [2] or by RT-PCR in reticulocytes (Table 1). However, it was detected in the nucleated dividing erythroleukaemic cell line K562. ACSL1_v2 (F-Gate) and v3 (no Gate) were detected in erythroid progenitor cells but v1 (Y-Gate) was not. ACSL6_v1 and v2 were found in both precursors cells and reticulocytes but v3 was not. Perhaps, in early stages of erythroid differentiation, ACSL1 might have function(s) not requiring a Gate domain while ACSL6 must have both types. ACSL6 variant 3, which lacks the Gate domain and has been isolated from human testis, was not detected in the tissues we surveyed. The existence of ACSL6_v3 [alternative splice site in exon 3 and exclusion of both Ex(Y) and Ex(F)] is supported by the fact that variant 3 of ACSL1, variant 2 [GenBank:AAH57770] of SLC27A2 [GenBank:NP_003636.1] (Figure 6), and short form 1 of GR-LACS [9] also lack the Gate-domain region. Moreover, an identical alternative splicing event affecting exon 3 was identified in cDNA isolated from bone marrow of an individual with acute eosinophilic leukemia [10] and a patient with atypical CML [11].

In the plasma membrane of adult red cells, the function of ACSL6 is activation of long-chain acyl groups for remodeling of lipids and acylation of proteins. In contrast, the predominant functions of ACSL members found in internal membranes are fatty acid degradation and *de novo *lipid synthesis. Not counting glycolipid species or other minor lipids, more than 250 different phospholipid molecular species have been characterized in the membrane of the red cell [12]. The different products of *ACSL6 *appear to be in a dimeric state, thus formation of hetero-dimeric complexes could produce enough variety to account for the specificity and selectivity in fatty acid activation observed in this membrane. Studies of the rat homologue indicate that ACSL6 is preferentially involved in metabolism of docosahexaenoic acid, a poly-unsaturated fatty acid highly enriched in the nervous system [13]. We also reported that human ACSL6 is expressed in brain tissue [2], which may suggest an important role of this protein in the plasma membranes of neuronal cells that experience rapid phospholipid turnover.

## Conclusion

We have identified several novel forms of acyl-CoA synthetase long-chain members that differ in their fatty acid Gate domain, and provided evidence that swapping or deletion of this domain appears to be conserved in all mammalian ACSL members 1 and 6. We propose that a Phe to Tyr substitution is the structural reason for the conserved alternative splicing that affects these motifs. This mechanism may lead to enzymes with different substrate specificity necessary to maintain the complex fatty acid composition of membranes.

## Methods

### DNA manipulations

PCR amplifications were performed from cDNAs of CD34 positive progenitors, K562 cells, fetal blood cells, reticulocytes and placenta [2]. Amplification of the Gate-domain regions of *ACSL1 *and *ACSL6 *were performed with the following set of primers:

Hs-ACSL6-902 (5'-ACTGTGGCCAAGAGAATCACCAGGCTCC-3'), Hs-ACSL6-1266/rev (5'-GTACATCCGGTTCAGCAGTCGTGGGACC-3') and Hs-ACSL1-937 (5'-CCTGGGAAGAGCCAACAGACGGAAGCCC-3'), Hs-ACSL1-1294/rev (5'-CCGGTTCAGCAGTCTTGGAACCACGGGG-3').

The PCR amplifications were performed with 1 μl of cDNA with Expand High-Fidelity Taq polymerase according to the manufacturer instructions (Roche). The annealing temperature was 66°C for *ACSL1 *and 64°C for *ACSL6*. The reaction conditions were as follows: one cycle at 95°C for 2 min, 40 cycles at 94°C for 30 s, 64°C or 66°C for 60 s, 72°C for 30 s, and one cycle at 72°C for 10 min. The PCR fragments (≈350 bp), isolated and purified from 1.5% agarose gels, were cloned with the Zero-Blunt PCR cloning kit (Invitrogen). Single colony transformants were selected on Kanamycin 50 μg/ml Luria-Broth plates. Two μl of an overnight grown culture were used directly for PCR amplification of the insert. Amplifications were done using M13(-20)fwd and M13(-27)rev primers with Promega Taq polymerase in buffer B according to the manufacturer instruction. First, 2 μl of cells were lysed and proteins denatured at 95°C for 10 min. in 50 μl of water. Then, 50 μl of doubled-concentrated PCR reagent mixture (25 ng of each primer, 200 μM dNTPs, 1 unit Taq, buffer B 2×) was added, and amplification reactions were done at 94°C for 60 s, 25 cycles at 94°C for 30 s, 47°C for 30 s, 72°C for 30 s, and one cycle at 72°C for 10 min. In each case, the single PCR product was isolated with the PCR purification kit of Qiagen and was sequenced. 5'-RACE PCR of human Leukaemia, Chronic Myelogeneous Marathon-Ready cDNA (K562 cells) (Clontech Laboratories, Inc.) for isolation of ACSL1_v1 was performed with primer Hs. ACSL1-1294/rev, and AP1 according to the manufacturer instructions. Full-length RACE PCR of reticulocyte cDNA for isolation of ACSL6_v5 was performed as previously described [2].

### Expression and immunodetection of ACSL6

ACSL6 isoforms were cloned by PCR into vector pET28a (Novagen) with a unique in-frame hexahistidine tag at the N-terminus, and were transformed into *E. coli *host BL21(DE3) cells. Cloning of ACSL6_v2 has been previously described [2]. Coding sequence of variant 3 was obtained by PCR amplification of the cDNA clone MGC:48352 [GenBank: BC047453] obtained from American Type Collection Center. The 1,889 bp fragment was amplified by a primer set introducing a restriction site for *Nhe*I at the 5' end and *Hind*III at the 3' end, and was then cloned with the Zero-Blunt PCR cloning kit of Invitrogen according to the manufacturer instructions, to yield plasmid pFK128. Forward primer was L6-v3-Nhe (5'-ACTATAGCTAGCCAGACACAGGAGATCCTG-3') and the reverse primer was L6-v3-Hind (5'-CTATGCAAGCTTCACATGGAGATTGAGTA-3') (*Nhe*I and *Hind*III sites are underlined). The 1.9 kbp *Nhe*I-*Hind*III fragment of pFK128 was cloned in frame into pET28a opened by *Nhe*I and *Hind*III, to yield plasmid pFK129. ACSL6_v2 (pKTM99) and ACSL6_v3 (pFK129) plasmids were transformed into chemically competent BL21(DE3) cells and selected on kanamycin (30 μg/ml) Luria-Bertani plates. Cells were grown at 37°C in liquid medium with kanamycin to an O.D.600 nm of 0.5 to 0.6 and grown for another 2 hours in the absence or presence of 100 μM of IPTG. Cells were chilled on ice and harvested by centrifugation at 6,000 g for 5 min at 4°C. The medium was discarded and pellets were quickly frozen in dry-ice and stored at -80°C. Total protein extracts were obtained by sonication of thawed pellets suspended in Buffer A [Tris-HCl 50 mM, pH 8.0; EDTA 5 mM; NaCl 0.3 M; DTT 5 mM; PMSF 0.1 mM] using 3 pulses of 20 s on ice. The lysates were cleared by centrifugation at 8,000 g for 20 min at 4°C. Most of the fusion protein was lost during this step and was found in inclusion bodies. The same was true when cells were grown at 25°C [8]. Less than 10% was still present in the cleared lysate. To prepare the membrane and soluble fractions, total extracts were subjected to centrifugation at 100,000 g for 60 min at 10°C. The supernatant represented the soluble fraction. The pellet was suspended in buffer A and represented the membrane fraction. Protein samples, about 10 μg, were denatured for 20 min at 37°C in 20 μl SDS-PAGE loading buffer 1× and were loaded in each lane of a SDS-PAGE 7.5% gel. The high molecular band (130 kDa) (Figure 3A), also observed on 2D-gels, was not detected when the protein samples were boiled before loading and/or when the samples were further diluted in SDS-PAGE loading buffer. Rat ACSL6-FLAG fusion proteins [8] showed the same migration pattern that human ACSL6-His tag proteins do under these conditions. Immuno-detection was performed with a commercial anti-histidine HRP-conjugated antibody (INDIA-HisProbe-HRP antibody, Pierce) (Figure 3A) and with a peptide-raised ACSL6 antibody (Figure 3B) detected with a HRP-conjugated goat anti-rabbit IgG (Pierce). Each antibody was diluted a thousand fold. HRP detection was performed with ImmonoPure DAB (Pierce) according to the manufacturer instructions.

### Sequence analysis

Sequence alignments were generated with MUTALIN [14]. Sequences were retrieved from RefSeq [15,16], and GenBank databases. Exon and intron definitions of *ACSL *genes were obtained using Evidence Viewer, and Model Maker available at the Map Viewer page of each gene.

### Accession numbers

The nucleotide sequences for ACSL1 variant 1, ACSL1 variant 3, ACSL6 variant 4 and ACSL6 variant 5 have been deposited in the GenBank database under GenBank Accession Number DQ083029, DQ083028, DQ083030 and DQ083031, respectively.

## Authors' contributions

ES conceived the study, its design, and carried out the experiments. FK participated in the conception of the study and interpretation of data. ES and FK wrote the manuscript.

## Figures and Tables

**Table 1:** Transcript variants for *ACSL1 *and *ACSL6 *identified in various tissues. Variants for ACSL1 and 6 were identified from cDNAs by PCR amplification of the Gate-domain region (see Methods). The number of clones representing each of the variants found in the population of sequenced clones is given as a ratio and is shown under each respective variant.

	***ACSL1***^*1*^	***ACSL6***^*2*^
**CD34^+ ^cells**	v2 – v3 (34/35 – 1/35)	v1 – v2 (19/22 – 3/22)
**Reticulocytes**	not detected	v1 – v2 – v4 – v5 (3/8 – 4/8 – 1/8) – (2/4)^*3*^
**K-562 cells**	v1 – v2 – v3 (1/11)^*3 *^– (29/30 – 1/30)	v1 – v2 – v4 (24/26 – 1/26 – 1/26)

^*1*^ACSL1_v1 was found in placenta (1/10). ACSL1_v2 was found in fetal blood liver (11/11) and placenta (9/10).

^*2*^ACSL6_v3 could not be found by PCR screening of an additional 38 clones.

^*3*^Clones identified by RACE-PCR in an independent experiment.

**Table 2:** Human acyl-CoA synthetase long-chain family member isoforms.

Gene	**Y-Gate** **D-x_4_-Y-LPLAH-x_2_-E**	**F-Gate** **D-x_4_-F-LPLAH-x_2_-E**	**No Gate**
***ACSL1***	variant 1/DQ083029	variant 2/NM_001995.2	variant 3/DQ083028
***ACSL3***	variant 1/NM_004457.3 variant 2/NM_203372.1		
***ACSL4***	variant 1/NM_004458.1 variant 2/NM_022977.1		
***ACSL5***	variant 1/NM_016234.3 variant 2/NM_203379.1 variant 3/NM_203380.1		
***ACSL6***	variant 1/NM_015256.2	variant 2/NM_001009185.1 variant 4/DQ083030 variant 5/DQ083031	variant 3/BC047453.1

The numbers listed represent the GenBank accession numbers of the respective spliced transcript variants.
